# Possible Signaling Pathways Mediating Neuronal Calcium Sensor-1-Dependent Spatial Learning and Memory in Mice

**DOI:** 10.1371/journal.pone.0170829

**Published:** 2017-01-25

**Authors:** Tomoe Y. Nakamura, Shu Nakao, Yukako Nakajo, Jun C. Takahashi, Shigeo Wakabayashi, Hiroji Yanamoto

**Affiliations:** 1 Department of Molecular Physiology, National Cerebral and Cardiovascular Center Research Institute, Suita, Osaka, Japan; 2 Laboratory of Neurology and Neurosurgery, National Cerebral and Cardiovascular Center Research Institute, Suita, Osaka, Japan; 3 Department of Neurosurgery, National Cerebral and Cardiovascular Center Hospital, Suita, Osaka, Japan; Universitatsklinikum Wurzburg, GERMANY

## Abstract

Intracellular Ca^2+^ signaling regulates diverse functions of the nervous system. Many of these neuronal functions, including learning and memory, are regulated by neuronal calcium sensor-1 (NCS-1). However, the pathways by which NCS-1 regulates these functions remain poorly understood. Consistent with the findings of previous reports, we revealed that NCS-1 deficient (*Ncs1*^*-/-*^) mice exhibit impaired spatial learning and memory function in the Morris water maze test, although there was little change in their exercise activity, as determined via treadmill-analysis. Expression of brain-derived neurotrophic factor (BDNF; a key regulator of memory function) and dopamine was significantly reduced in the *Ncs1*^*-/-*^ mouse brain, without changes in the levels of glial cell-line derived neurotrophic factor or nerve growth factor. Although there were no gross structural abnormalities in the hippocampi of *Ncs1*^*-/-*^ mice, electron microscopy analysis revealed that the density of large dense core vesicles in CA1 presynaptic neurons, which release BDNF and dopamine, was decreased. Phosphorylation of Ca^2+^/calmodulin-dependent protein kinase II-α (CaMKII-α, which is known to trigger long-term potentiation and increase BDNF levels, was significantly reduced in the *Ncs1*^*-/-*^ mouse brain. Furthermore, high voltage electric potential stimulation, which increases the levels of BDNF and promotes spatial learning, significantly increased the levels of NCS-1 concomitant with phosphorylated CaMKII-α in the hippocampus; suggesting a close relationship between NCS-1 and CaMKII-α. Our findings indicate that NCS-1 may regulate spatial learning and memory function at least in part through activation of CaMKII-α signaling, which may directly or indirectly increase BDNF production.

## Introduction

Spatial learning and navigation are critical to the survival of non-sessile animals. Extensive research has documented that such higher-order brain functions are associated with intracellular Ca^2+^ regulation, which plays major roles in both neurotransmitter release and synaptic plasticity through processes such as signal transduction, gene expression, and ion channel activity. For example, Ca^2+^/calmodulin-dependent protein kinase II (CaMKII) has been proposed as a key molecule in the mediation of learning and memory processes through potentiation of Ca^2+^-permeable ion channels [1] and phosphorylation of AMPA-type glutamate receptors, resulting in increased excitatory postsynaptic currents [2].

A close relationship between intracellular Ca^2+^ signaling and a number of neurotrophic factors such as brain-derived neurotrophic factor (BDNF), glial cell line-derived neurotrophic factor (GDNF), and nerve growth factor (NGF) exists [3–5]. BDNF, the most abundant of these factors, has an established role in promoting the differentiation and maturation processes of developing neurons [6]. Moreover, BDNF positively regulates synaptic transmission and plasticity in mature neurons [7], thereby contributing to learning and memory formation [8]. Research has indicated that transcription of the BDNF gene increases in response to intracellular Ca^2+^ elevation [3,9]. Dopamine modulates the transcription of a variety of genes, thereby promoting neuronal differentiation and survival [10] and long-term synaptic plasticity [11]. Dopamine exerts its actions through dopamine receptor types 1–5. Emerging evidence suggests that a positive relationship exists between BDNF and dopamine signaling [12], and that this interaction appears to be mediated by a Ca^2+^-dependent cascade [13].

The various actions of Ca^2+^ are mediated by a large family of Ca^2+^ sensor proteins that accomplish their target functions via spatiotemporal activation. Neuronal Ca^2+^ sensor-1 (NCS-1) is an EF-hand Ca^2+^ binding protein that has been implicated in neuronal functions such as synaptic transmission, short- and long—term synaptic plasticity [14–18], and neuronal survival [19]. NCS-1 has many downstream targets that account for its functional diversity, binding to and regulating voltage-gated K^+^ channels [20]; P/Q-type Ca^2+^ channels [21]; inositol 1,4,5-trisphosphate receptors [22–24]; phosphatidylinositol 4-kinase III-β [25]; and dopamine type-2 receptors (D2R) to preserve D2R signaling [26]. Some of these pathways also influence Ca^2+^ signaling [21,23–25]. In addition, NCS-1 has been reported to have an important role in higher-order functions such as learning and memory, as demonstrated by studies utilizing genetic deletion of NCS-1 in *C*. *elegans* [27]. In mice, overexpression of NCS-1 promotes rapid acquisition of spatial memory [17], whereas downregulation of NCS-1 levels by siRNA causes a deficit in self-directed exploration learning bonus [28]. Furthermore, NCS-1 deficits result in anxiety and depressive-like behavior, as well as impairments in non-aversive memory, in mice [29]. NCS-1 deficiency also decreases motivation-related activity in the nucleus accumbens [30]. Thus, NCS-1 influences neurophysiology, possibly through a combination of protein interactions [17,27,28]. However, the detailed molecular mechanisms by which NCS-1 regulates learning and memory remain largely unexplored.

In the present study, we utilized a modified Morris water maze (MWM) test to analyze the behavior of NCS-1 knockout mice. Consistent with the findings of previous reports, we observed that NCS-1 regulates spatial learning and memory formation. We subsequently investigated downstream pathways, including the involvement of neurotrophic factors and intracellular Ca^2+^-dependent signals. Our data suggest that the CaMKII pathway is an important mediator of NCS-1-regulated neurophysiology.

## Materials and Methods

### Animals

This study conforms to criteria outlined in the National Institutes of Health (NIH) Guidelines for the Care and Use of Laboratory Animals. Animal care and experimental procedures followed the Animal Welfare Committee guidelines and were approved by the institutional review board of the National Cerebral and Cardiovascular Center Research Institute (approval reference number: 16072). Efforts were made to minimize the number of animals used and their suffering. NCS-1 knock-out (KO/*NCS1*^*-/-*^) mice were generated via homologous recombination by Andreas Müllemann and colleagues (2005) from the Université de Genève, and Horst Bluethmann and colleagues from the Hoffmann La-Roche (Switzerland), as previously described [29]. The procedures used to create this mouse strain are available at the MGI database (accession # 3525199). Frozen embryos were rederived by Charles River Laboratory through Dr. Andreas Jeromin. Heterozygosity (*Ncs1*^*+/−*^) was maintained in mouse colonies. C57BL/6-NCR mice were backcrossed at least twenty times. Mice were genotyped by PCR analysis of genomic DNA and Western blotting, as previously described [23]. We used 5–7-week-old KO and wild-type (WT) littermate mice for most experiments. For the experiments using fetal mice (such as for cultured neurons), we used age-matched C57BL/6-NCR mice (Japan SLC, Inc. Hamamatsu, Japan). Mice were placed in a temperature and humidity-controlled (23–25°C, 50%) room under a 12-h light/dark cycle (lights on 7:00 AM) and had free access to food and water throughout the study. Mice were anesthetized with isoflurane (1–2% for maintenance; 3% for induction) in air administered using an anesthetic vaporization instrument (MK-A110D, Muromachi Kikai Co. Ltd.) prior to all but the behavioral experiments.

### Morris water maze (MWM) test

We conducted a modified MWM test as previously described [31] using a 64 cm × 91 cm pool in which a 10 cm ×10 cm platform was placed at a fixed position. The pool was filled with opaque water that had been prepared using a non-toxic agent, which covered the platform surface up to a height of 2 cm. The water temperature was maintained at 25°C during the procedure. Mice were inserted into the pool at the same point every time, which facilitated the learning process for mice to locate the platform. Each mouse performed 4 trials per day over 5 consecutive days, without any prior training. A successful escape was defined as observation of the mouse standing on the platform. We recorded the time required for the mouse to escape onto the platform as the escape latency. The cut-off time was set at 300 s for each trial. Mice that failed to reach the platform within 300 s were removed from the water, and their escape latency was recorded as 300 s. Thus, the learning procedure performed on days 2–5 was exactly the same as that performed on day 1, which was part of our design to enhance the consolidation process that occurred between days 1 and 2. In each trial, we analyzed the escape latency and total path length required to navigate to the platform using a video-tracking system (Smart; Panlab, Barcelona, Spain). On day 5, we performed a 1-min probe test during which the platform was removed from the pool. The number of times mice crossed the area where the platform had been located previously was recorded in order to assess long-term spatial memory.

### Treadmill exercise tolerance test

We used 6-week-old WT (weight: 21.0–22.6 g) and KO (weight: 19.4–23.1 g) male littermate mice (p>0.05). Prior to the test of their running ability, mice were first acclimated to a treadmill with 10 running lanes (MK680C, Muromachi Kikai Co. Ltd.) for three days (one 15-min running trial/day). Prior to testing, tail blood samples were taken for lactate concentration measurements. The slope and speed of the treadmill, as well as running time, were gradually increased as follows: 10°, 10 m/min, 5 min; 10°, 15 m/min, 5 min; 10°, 20 m/min, 60 min; 15°, 20 m/min, 30 min; 20°, 20 m/min, 30 min; 25°, 20 m/min, 30 min. When the rest due to exhaustion continued for more than 30 s, the running protocol was terminated, and a blood sample was taken within 1 min to assess the blood lactate concentration (Lactate Pro, ARKRAY, Inc.). The total running distance was calculated for each mouse as an indication of its running ability.

### Primary cultures of neurons and immunocytochemistry

Primary cultures of cerebral neurons were generated from WT and KO mice at embryonic day 18, as previously described [19]. In brief, cerebrum tissue containing cortex and hippocampus was isolated from the whole brain, cut into small pieces, and digested for 10 min at 37°C in a 20-U/ml papain solution containing 0.002% DNase I (Worthington Biochemical Corp., Lakewood, NJ, USA). After titration of the enzymatic activity, the cells were mechanically dissociated by several passages through pipette tips. After centrifugation, the cells were re-suspended in neurobasal medium supplemented with B27 trophic factors (both from Thermo Fisher Scientific, Waltham, MA, USA) [32]. The cells were plated onto 1% polyethylenimine-coated glass-bottom dishes at a density of 5×10^4^ cells/cm^2^ for immunofluorescence analysis.

At 3–4 days after plating, the cerebrum neurons obtained from WT or KO mice in the primary culture were either treated or left untreated. They were fixed with 10% neutral buffered formalin, permeabilized with 0.2% Triton X-100, and blocked with 5% bovine serum albumin (Sigma-Aldrich, Tokyo, Japan). They were then incubated for 1 hour with primary antibodies, including mouse monoclonal anti-NCS-1 antibody (1:100 dilution, BD Biosciences, Franklin Lakes, NJ, USA), rabbit polyclonal anti-microtubule associated protein (MAP2) antibody (1:100 dilution, Abcam, Cambridge, MA, USA) as neuronal markers; rabbit monoclonal anti-glial fibrillary acidic protein (GFAP) antibody (1:100 dilution, Abcam) as an astrocyte marker, or anti phospho-CaMKII-α (Thr286) antibody (1:100 dilution, Cell signaling). This procedure was followed by incubation with fluorescent-conjugated secondary antibodies. After extensive washing with phosphate-buffered saline (PBS), cell images were obtained using an inverted microscope (Olympus 1X81, equipped with 60×/1.42 oil immersion objective lens, Olympus Optical Co., Tokyo, Japan) attached to a confocal laser-scanning unit, and FV10-ASW imaging software (V 4.02).

### Histology

The brains of WT and KO mice were quickly removed, flash frozen with liquid nitrogen, and maintained at -80°C until processing. Thereafter, 10-μm-thick cryosections were prepared at -15°C using a Leica CM1850 cryostat (Leica Biosystems, Tokyo, Japan). Immunohistochemistry was performed using the labeled biotin-streptavidin method, as previously described [19]. In brief, slides were fixed and pretreated with 0.3% (v/v) hydrogen peroxide for 15 min at room temperature (25±3°C). They were then blocked with 1% goat serum, followed by incubation at 4°C overnight with mouse monoclonal anti-NCS-1 antibody (1:100 dilution, BD Biosciences). The slides were then incubated with a biotinylated secondary antibody and an HRP-conjugated streptavidin-biotin complex (R.T.U. Vectastain Universal Elite ABC Kit; Vector Laboratories, Inc., Burlingame, CA, USA). The colored reaction product was developed using a DAB solution (Vector Laboratories, Inc.). The sections were lightly counterstained with hematoxylin to visualize the nuclei. Images were acquired using a digital camera (DP-72, Olympus Optical Co., Tokyo, Japan) equipped with image filing software (DP2-BSW, Olympus Optical Co.).

For morphometrical analysis, 4-μm-thick cross-sections were prepared from the formalin-fixed paraffin-embedded brains and subsequently stained with hematoxylin/eosin to visualize the brain structure. Images were selected from six different regions of the brain sections, which contained the greatest possible area of the hippocampus and dentate gyrus. All nuclei of the granule cells in each image were counted to determine the cell number. To assess levels of apoptosis in the tissue sections, we performed terminal deoxynucleotidyl transferase biotin-dUTP nick-end labeling (TUNEL). The sections were lightly counterstained with methyl green.

### Western blotting

Proteins were extracted from mouse brains and subjected to immunoblot analysis, as previously described [19]. In brief, brains were solubilized in a urea-containing lysis buffer (2 M urea, 10 mM NaHCO_3_, 1 mM EDTA-Na, and 2% SDS) supplemented with a protease inhibitor cocktail (Roche, Basel, Switzerland). Protein concentrations were determined using a BCA assay kit (Thermo Fisher Scientific). Equal amounts of protein were separated onto NuPAGE denaturing bis-tris SDS-polyacrylamide gels (Thermo Fisher Scientific), transferred to polyvinylidene difluoride membranes, and subjected to immunoblotting. To determine levels of NCS-1 expression, rabbit polyclonal anti-NCS-1 antibody (1:1000 dilution) [19] or mouse monoclonal anti-NCS-1 antibody (1:100 dilution, BD Biosciences) was used. Phospho-CaMKII-α (Thr286) antibody (1:100 dilution, Cell signaling) was also used. Anti-GAPDH (glyceraldehyde 3-phosphate dehydrogenase) antibody (1:1000 dilution, Merck Millipore, Billerica, MA, USA) served as the loading control. Band density was quantified using Image-Pro Plus Version 7 (Media Cybernetics, Rockville, MD, USA).

### Measurement of neurotrophic factors and dopamine levels

The levels of BDNF, GDNF, and NGF were measured as previously described [31]. The PBS-perfused cerebral cortex was excised from each mouse and homogenized. Protein levels were measured using a two-site sandwich ELISA (Emax Immunoassay System, Promega, Madison, WI, USA). Dopamine levels in the mouse brains were measured using a Dopamine ELISA kit (KA1887, Abnova Corp, Taiwan), according to the manufacturer’s instruction. The protein concentration in each sample was measured using a BCA protein assay kit (Thermo Fisher Scientific).

### Transmission electron microscopy

Brains were removed from WT and KO mice and immersed in a fixative solution containing 2.5% glutaraldehyde in PBS for 2 hours, followed by three times washing in 0.1 M phosphate buffer. They were then postfixed in cold 1% osmium tetroxide solution for 2 hours. After dehydration in a series of graded ethanol solutions, the sections were embedded in epoxy resin. Serial ultra-thin sections (100 nm thick) were cut using an ultra-E microtome (Leica Microsystems, Wetzlar, Germany) and mounted on uncoated 200-μm mesh copper grids. They were then stained with uranyl acetate/lead citrate and analyzed using an electron microscope (H-7100, Hitachi High-Technologies Corp., Tokyo, Japan). Digitized images were processed using Photoshop CS3 (Adobe, San Jose, CA, USA)

For large dense-core vesicle (LDCV) analyses, the LDCVs were identified according to size (>100 nm) and electron density based on the presence of various neurotransmitter peptides. The number of LDCVs was counted in the presynaptic terminals of hippocampal CA1 neurons in WT and KO mouse brains and represented as the ratio of the number of LDCVs to the number of synapses.

### Treatment with high voltage electric potential (HELP)

Mice were treated with HELP as previously described [33]. Briefly, each mouse cage was composed of 2-mm-thick acryl boards and housed five to six mice. A mattress-type HELP supplier, connected to the HELP generator (an AC-transformer; 60 Hz), was placed under the cage. The grounded counter electrode was located 20 cm below the HELP supplier. The HELP area, comprising the HELP supplier, cage, and mice, was electrically insulated from its surroundings by means of an insulating table, so that the HELP-exposed mice only made indirect contact with the 0 V surface (ground) via room air. The HELP generator increased the electric potential at the HELP applying surface (the floor of the cage) to 0 V (untreated control) or 5.8 kV (measured by Statiron-DZ3, SSD Electrostatic Ltd., Tokyo, Japan) for 5 h a day over 3 consecutive weeks. Under these settings, the electric field generated above the HELP supplier in the absence of the mice was 0 V or 24–25 kV/m, respectively.

### Statistical analysis

The differences between the two groups were analyzed using unpaired Student’s *t*-tests. Groups with one variable were analyzed using one-way ANOVA (parametric) followed by Tukey's multiple comparison test. For comparison among groups with two variables, two-way ANOVA with or without repeated measures were used, followed by the Holm-Sidak method for all *post hoc* pairwise multiple comparisons. Group data are presented as mean ± standard error of the mean (SEM). For all comparisons, statistical significance was set at p<0.05. Asterisks denote the following: * P <0.05, ** P <0.01.

## Results

### Spatial learning and memory is impaired in *Ncs1*^-/-^ mice with little change in motor activity

**Fig 1:**
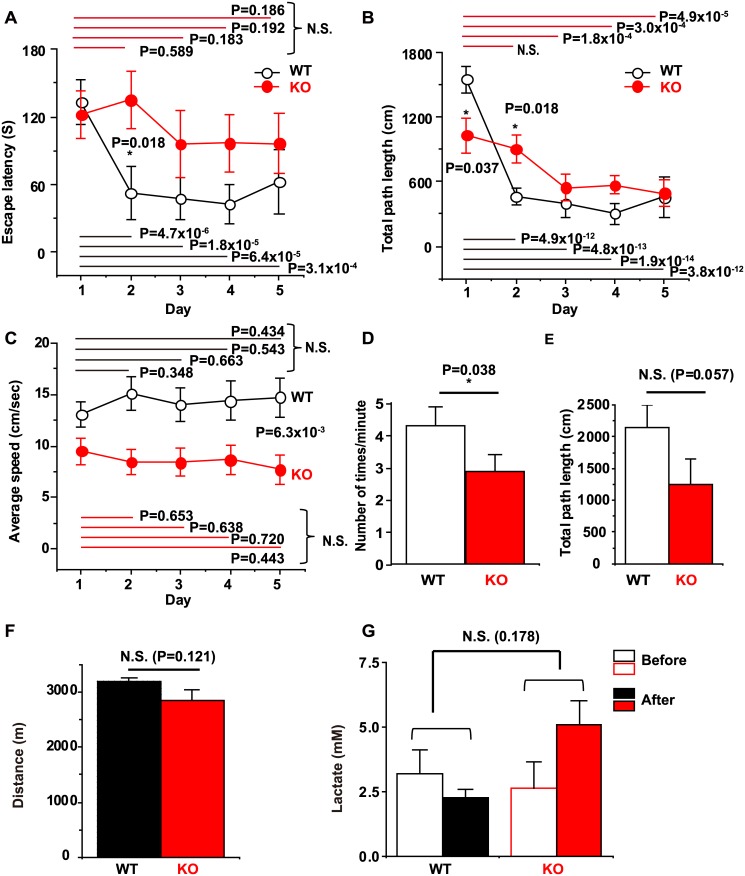
Analysis of Morris water maze and treadmill test results in wild-type (WT) and *Ncs1*^-/-^ (knock-out [KO]) mice. Escape latency (A), total path length (B), and average swimming speed (C) to the hidden platform during trials over 5 consecutive days. KO mice exhibit inferior performance in escape latency, total path length, and average swimming speed [two-way ANOVA with repeated measures followed by a pairwise multiple comparison using a Holm-Sidak test: DF = 4, F = 5.83, P = 3.4x10^-4^ for days, and DF = 1, F = 2.09, P = 0.162 for genotype in (A), and DF = 4, F = 27.5, P<1x10^-9^ for days, and DF = 1, F = 0.497, P = 0.488 for genotype in (B), and DF = 4, F = 0.175, P = 0.951 for days, and DF = 1, F = 9.13, P = 6.2x10^-3^ for genotype in (C)]. The P values obtained by all pairwise comparison are indicated in the figures. (D) The result of probe test indicating that the number of times the mice crossed the area where the platform had been located previously in one minute. (E) Total path length measured on the first trial of the first day to evaluate exploratory activity. Statistical analysis was performed using Student’s *t*-test. P = 0.038 and P = 0.057 for (D) and (E), respectively. (F and G) Exercise capacity evaluated by performing a running tolerance test using the treadmill apparatus and measurement of blood lactate levels. There was no statistically significant difference between WT and KO mice in either the run distance (Fig 1F, P = 0.121, by Student’s *t*-test), or lactate accumulation (two-way ANOVA, DF = 1, F = 1.993, P = 0.178). Data are presented as mean ± standard error of the mean; n = 12 mice/each group; NCS-1: neuronal calcium sensor-1.

To analyze the mechanisms underlying NCS-1-regulated spatial learning and memory function in mice, we performed a modified Morris water maze (MWM) test. In WT mice, the escape latency to the hidden platform as well as the total path length in each session continued to decrease after the first trial, with significant differences observed between day 1 and days 2–5 (P<0.05), indicating that mice had learned the location of the platform on the second day (Fig 1A and 1B). In contrast, KO mice exhibited no considerable improvement in escape latency (i.e. there was no statistical difference between results on day 1 and days 2–5, Fig 1A). Similarly, KO mice exhibited less improvement in total path length: No significant difference in total path length was observed between day 1 and day 2, although a significant difference was noted between day 1 and days 3–5, with much larger P values than those observed for WT mice (Fig 1B). A significant difference in both escape latency and total path length was observed between WT and KO mice on day 2 (P = 0.018 each). In addition, the results of the probe test revealed that KO mice exhibited impairments in long-term memory (Fig 1D). To examine whether motor activity was also affected, we calculated the average swimming speed (Fig 1C), which was significantly lower in KO mice (P = 6.3×10^−3^), suggesting that the increased escape latency of KO mice may be the result of lower motor activity. To compare the exercise capacity of the mice more directly, we performed a running tolerance test using a treadmill apparatus where the slope and speed of the treadmill, as well as the running time, were gradually increased. As shown in Fig 1F, both WT and KO mice were able to run similar distance (P = 0.121 with Student’s *t*-test). Furthermore, there was no statistically significant difference in accumulation of blood lactate between the WT and KO group (two-way ANOVA, DF = 1, F = 1.993 and P = 0.178), indicating that KO mice have an exercise capacity comparable to WT mice. Taken together, these results suggest that KO mice exhibited impairments in spatial learning and memory function without major defects in motor activity.

### High expression of NCS-1 in cortical and hippocampal neurons in the mouse brain

**Fig 2:**
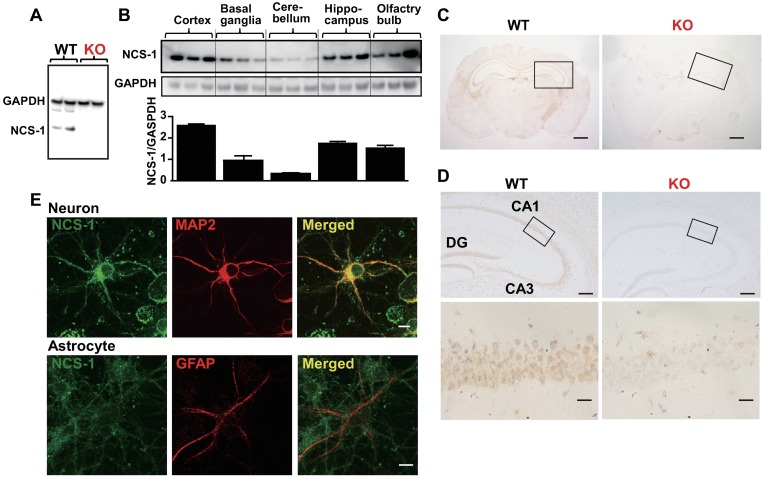
Localization of neuronal calcium sensor-1 (NCS-1) in the mouse brain. (A and B) Western blotting of brains from wild-type (WT) and *Ncs1*^**-/-**^ (knock-out [KO]) mice was performed with anti-NCS-1 monoclonal antibody to evaluate the specificity of antibody (A) and examine the localization of NCS-1 in different regions of brain (B). Anti-GAPDH antibody was used for normalization. (C) Cross-sections of the brains of 6-week-old WT and KO mice subjected to immunohistochemistry for NCS-1. The nuclei were counterstained with hematoxylin and visualized in blue. Scale bars = 1 mm. (D) Higher magnification images of the hippocampus in C. CA1: Cornu Ammonis 1; CA3: Cornu Ammonis 3; DG: dentate gyrus. Scale bars = 200 μm (upper panels) and 20 μm (lower panels), respectively. (E) Representative images of immunocytochemistry showing a MAP2-positive cortical neuron (red signals in upper panels) and a GFAP-positive astrocyte (red signals in lower panels), labeled with NCS-1 (green signals in upper and lower panels), isolated from the cortex of a WT mouse embryo (E18). Scale bar = 10 μm. MAP2: Microtubule-associated protein 2; GFAP: glial fibrillary acidic protein; GAPDH: glyceraldehyde 3-phosphate dehydrogenase.

Although KO mice exhibited impairments in learning ability, the weights of the whole brain, cortex, basal ganglia, hippocampus, cerebellum, and olfactory bulb were similar between WT and KO mice (Table 1). Moreover, we examined the expression levels and localization pattern of NCS-1 in different brain regions using a specific antibody against NCS-1, for which no signal was observed in KO brains (Fig 2A). Western blot analysis revealed that NCS-1 is expressed throughout the brain and enriched in the cortex, hippocampus, and olfactory bulb; moderately expressed in the basal ganglia, and barely expressed in the cerebellum (Fig 2B), consistent with our immunohistochemical data (S1 Fig). Immunohistochemical analysis also revealed that NCS-1 was highly expressed in hippocampal pyramidal and dentate gyrus granule neurons at the cell body as well as in dendrites (Figs 2C, 2D and S1), consistent with the findings of previous reports that used different antibodies [20,34]. To determine NCS-1 expression in specific cell types, we performed immunocytochemical analysis using cultured neurons and glial cells. Although NCS-1 was barely detected in glial cells, which were visualized as GFAP-positive cells (Fig 2E), it was strongly detected in neurons, particularly in the cell bodies and dendrites (detected as MAP2-positive cells in Figs 2E and S1).

**Table 1:** Body, whole-brain, and brain region weights.

	WT	KO	P value
Body weight (g)	23.4±0.58	24.3±0.98	0.228
Whole brain (mg)	480.8±5.84	479.0±5.14	0.409
Cortex (mg)	168.2±2.41	173.0±2.48	0.096
Basal ganglia (mg)	124.8±3.59	120.7±1.02	0.145
Hippocampus (mg)	44.0±1.15	46.3±1.23	0.098
Cerebellum (mg)	131.3±2.29	132.0±2.21	0.419
Olfactory bulb (mg)	43.8±0.54	44.7±1.17	0.267
Total brain/body weight (%)	2.06±0.08	1.98±0.07	0.241

The weights were obtained from 7-week-old wild-type (WT) and *Ncs1*^-/-^ (knock-out [KO]) mice (n = 9 mice/each group).

### Hippocampal structure is similar between WT and *Ncs1*^-/-^ mice

**Fig 3:**
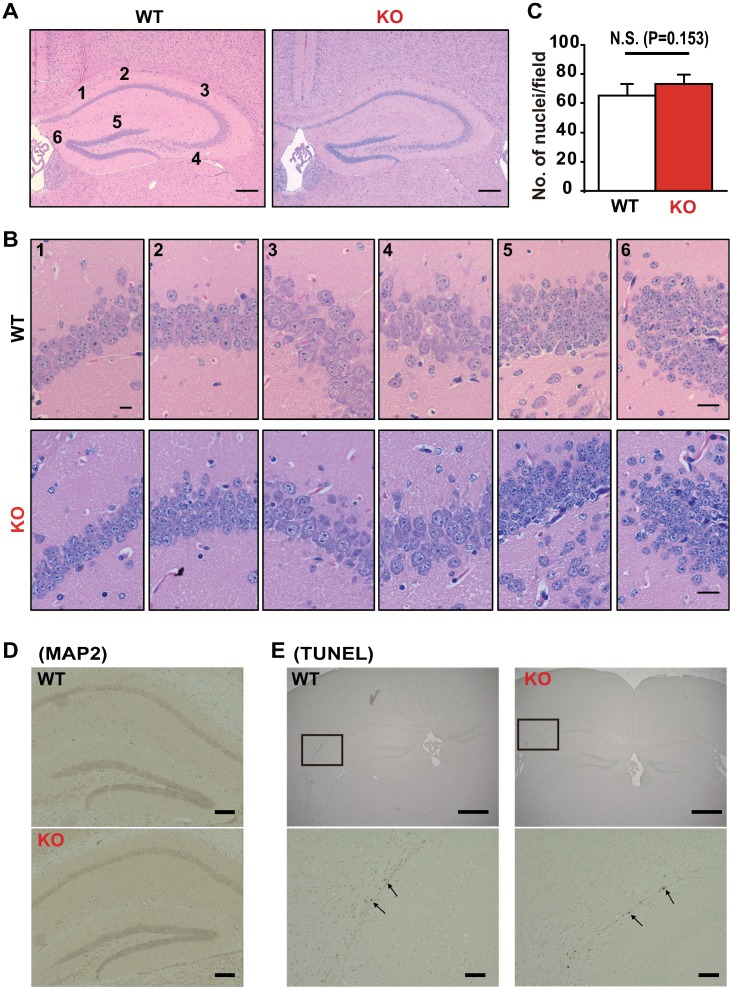
Morphology of mouse brains. (A) Hematoxylin and eosin (HE) staining of cross-sections of the hippocampus from 6-week-old wild-type (WT) and *Ncs1*^-/-^ (knock-out [KO]) mice. Scale bars = 200 μm. (B and C). We quantified cell number by counting the number of nuclei in six regions of the CA1, CA2, CA3 and DG (both the left and right side of the brain). Representative photographs are shown in (B) Scale bars = 20 μm. The average data for the number of nuclei obtained from the six regions in WT and KO mice (C). n = 6/group. There was no statistically significant difference between the WT and KO groups (P = 0.153). (D) Representative photographs of hippocampal cross-sections immunolabeled with the neuronal marker microtubule-associated protein (MAP2). Scale bars = 200 μm. (E) Representative photographs of a cross-section treated with terminal deoxynucleotidyl transferase biotin-dUTP nick end (TUNEL). The arrowheads indicate TUNEL-positive cells, suggesting apoptosis. Scale bars = 1 mm and 200 μm for upper and lower panels, respectively.

Next, we examined whether the hippocampal structure differed between WT and KO mice. Hematoxylin and eosin staining revealed no obvious histological alterations in the Cornu Ammonis (CA) areas or dentate gyrus in the KO brains when compared to WT brains (Fig 3A). The number of nuclei as an indicator of cell number in the CA areas and dentate gyrus were similar between WT and KO mice (Fig 3B and 3C). In addition, the MAP2 signals and apoptotic cell number did not markedly differ between the two groups (Fig 3D and 3E). These results suggest that NCS-1 deficiency does not affect the gross histological structure of the mouse hippocampus under basal conditions.

### BDNF levels and number of LDCVs in hippocampal neurons were decreased in the *Ncs1*^-/-^ mouse brain

**Fig 4:**
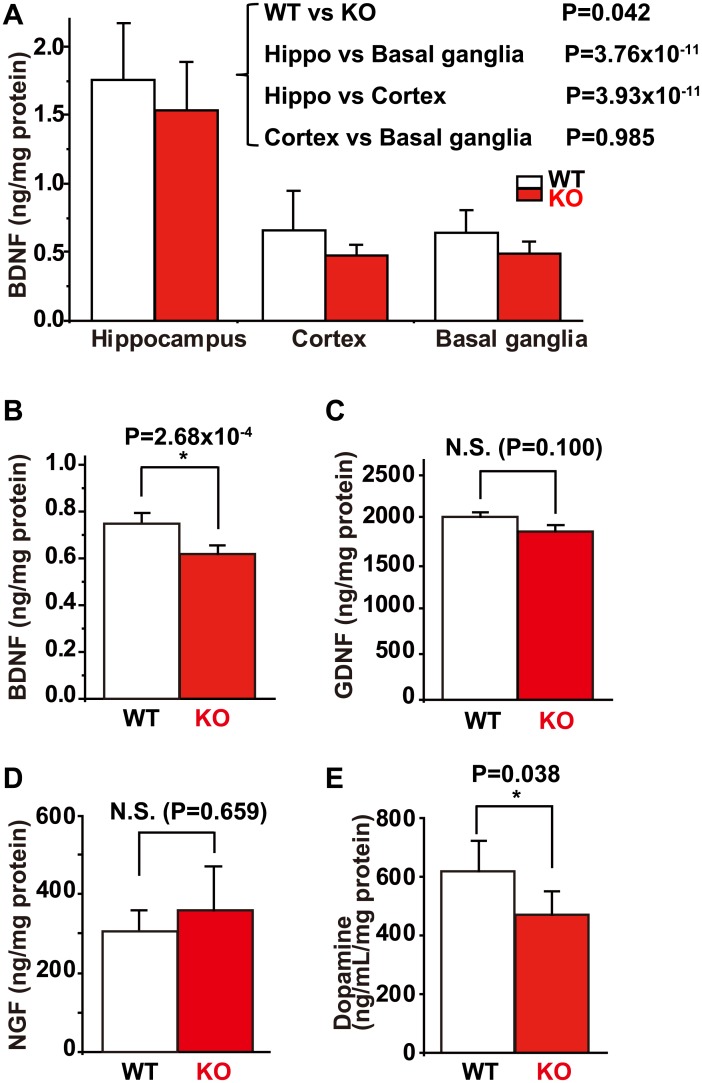
Levels of neurotrophic factors and dopamine in the mouse brain. (A) Average data for levels of brain-derived neurotrophic factor (BDNF) in the hippocampus, cortex, and basal ganglia in wild type (WT) and *Ncs1*^-/-^ (knock-out [KO]) mice. Two-way ANOVA indicated that there was a statistically significant difference in BDNF levels between the WT and KO groups (DF = 1, F = 4.512, P = 0.042), and between the different regions (DF = 2, F = 67.7, P<0.001), specifically between hippocampus and basal ganglia (P = 3.76×10^−11^), and between the hippocampus and cortex (P = 3.93×10^−11^), but not between the cortex and basal ganglia (P = 0.985), that was evaluated by Holm-Sidak multiple comparison procedure. (B) to (D) Average data for BDNF (B), glial cell line-derived neurotrophic factor (GDNF) (C), and nerve growth factor (NGF) (D) levels in the brains of WT and KO mice. A Student’s *t*-test analysis revealed that there was a statistically significant difference in BDNF levels in the forebrain between WT and KO mice (P = 2.68×10^−4^), but not in GDNF (P = 0.100) or NGF (P = 0.659) levels. (E) There was a statistically significant difference in levels of dopamine between WT and KO mice across the whole brain (P = 0.038). Data are presented as mean ± standard error of the mean, n = 6 for each group.

To investigate whether neurotrophic factors are involved in the NCS-1-dependent regulation of learning and memory, we analyzed the protein levels of BDNF, GDNF, and NGF in WT and KO mouse brains using ELISA assays. BDNF levels were markedly higher in the hippocampus as compared to other regions of the brain in both WT and KO mice (Fig 4A). In addition, two-way ANOVA indicated that, overall, there was a statistically significant difference in BDNF levels between WT and KO mice in the hippocampus, cortex, and basal ganglia. This result was consistent with findings obtained in the forebrain (which includes all of these regions), with significantly lower BDNF levels observed in KO mice (Fig 4B). However, there was no statistical difference when comparing the hippocampus, cortex, and basal ganglia between WT and KO mice. In contrast, the levels of GDNF and NGF were not significantly different between WT and KO brains (Fig 4C and 4D). Since NCS-1 interacts with D2R, we also examined dopamine levels, which were lower in the brains of KO mice (Fig 4E).

**Fig 5:**
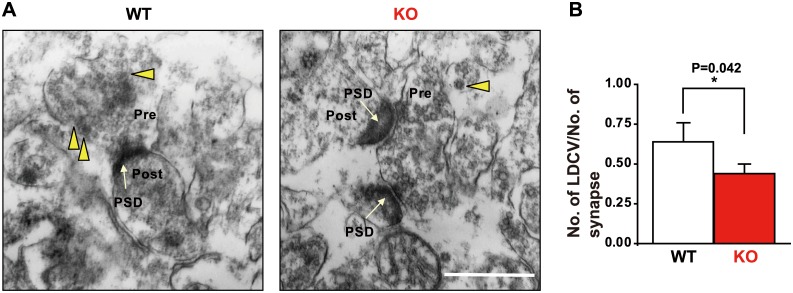
Ultrastructure of hippocampal CA1 neurons in wild-type (WT) and *Ncs1*^-/-^ (knock-out [KO]) mice. (A) Representative photographs visualized using transmission electron microscopy. Yellow arrowheads and white arrows indicate large dense core vesicles (LDCVs) and post synaptic densities (PSD), respectively. (B) Average data for the number of LDCVs per presynaptic terminal in WT and KO mice. Scale bar = 500 nm. Data are presented as means ± standard error of the mean; n = 82 and 87 synapses in the WT and KO group from 16 areas, respectively. The number of LDCVs per presynaptic terminal was lower in KO mice compared to WT mice (Student’s *t*-test, P = 0.042).

We examined the ultrastructure of neuron synapses in the hippocampus. Electron microscopy analyses revealed that the number of large dense core vesicles (LDCVs), from which neurotrophic factors including BDNF and catecholamines such as dopamine are released, in the presynaptic terminal of CA1 neurons of the hippocampus was significantly lower in the KO group than in the WT group (Fig 5).

## Effects of High Voltage Electric Potential (HELP) Treatment

**Fig 6:**
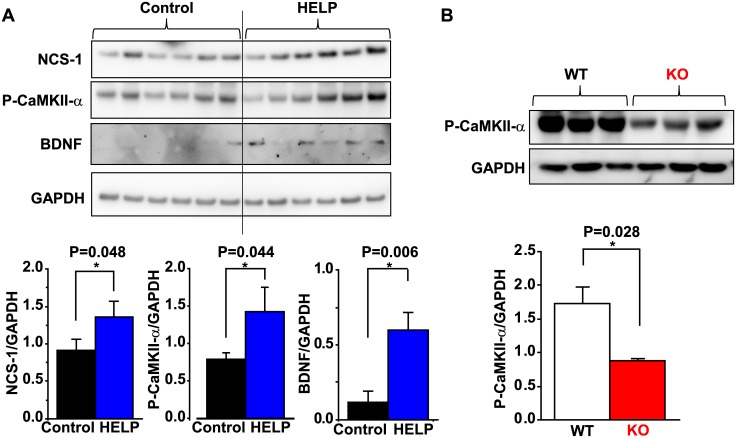
NCS-1 levels with or without high voltage electric potential (HELP) stimulation. (A) Representative Western blotting for neuronal calcium sensor-1 (NCS-1), phosphorylated Ca^2+^/calmodulin-dependent protein kinase-II alpha (P-CaMKII-α), brain-derived neurotrophic factor (BDNF), and glyceraldehyde 3-phosphate dehydrogenase (GAPDH) in the hippocampus obtained from wild-type (WT) mice subjected to HELP (5.8 kV, 5 hours a day, over 3 consecutive weeks). The average data for NCS-1/GAPDH, P-CaMKII-α, GAPDH, and BDNF/GAPDH are summarized. Data are presented as means ± standard error of the mean. n = 6 for each group. *P<0.05. (B) Representative Western blotting for P-CaMKII-α and GAPDH in the WT and KO mouse brain under basal conditions. Summarized data are also shown.

Since we previously observed that repeated HELP application increased levels of BDNF in the brain and enhanced spatial learning [33], we examined the involvement of NCS-1 in this process. Following HELP treatment, NCS-1 levels in the hippocampus were significantly increased compared to those of untreated controls (Fig 6A). Because BDNF expression is promoted by activation of CaMKII-α [35], we also examined whether levels of phosphorylated CaMKII-α expression in the hippocampus are differed between controls and HELP-treated mice. As expected, phosphorylated CaMKII-α levels were increased concomitantly with increased NCS-1 expression following HELP treatment (Fig 6A). Moreover, as previously observed [33], HELP treatment increased BDNF levels (Fig 6A). We also investigated whether phosphorylated CaMKII-α levels are altered in KO mice under basal conditions. Indeed, we observed that phosphorylated CaMKII-α levels were significantly decreased in mice of the KO group (Fig 6B), further suggesting a close relationship between NCS-1, CaMKII signaling, BDNF production, and spatial learning and memory function.

## Discussion

In the present study, we analyzed the specific signaling pathways by which NCS-1 contributes to spatial learning and memory in mice. Our data show that (1) NCS-1 deficiency results in impaired learning and memory without significant decreases in exercise activity or neural cell loss; (2) BDNF levels were decreased in the forebrains of KO mice relative to those of WT mice; 3) levels of phosphorylated CaMKII-α, a key mediator of memory, were significantly decreased in the brains of KO mice under basal conditions, 4) repeated HELP application, known to promote learning and memory function, also increased levels of NCS-1, phosphorylated CaMKII-α, and BDNF. These results suggest that NCS-1 activates Ca^2+^-dependent CaMKII-α signaling, which may directly and/or indirectly increase BDNF levels, and contributes to normal spatial learning and memory function in mice.

Analysis of MWM results indicated that both escape latency and total path length on days 2–5 in the WT group were significantly shorter than those on the first day, demonstrating that mice had learned the location of the platform by the second day. In contrast, the escape latency on days 2–5 or total path length on day 2 were not shorter for the KO group, demonstrating an inability of the KO mice to memorize the platform location, despite multiple learning opportunities. Since there was a significant difference in both escape latency and total path length between WT and KO mice on day 2 (P = 0.018), we concluded that the KO mice exhibited a deficit in spatial learning performance (as reported previously; [17,28–30]). Our data indicate that this difference in learning and memory ability, if any, is most apparent on day 2 of the spatial learning test. The reason is that the procedures of the first day are designed for achieving spatial memory, while the procedures from the third to the fifth days represent the repetition of the initial consolidation process that occurred between the first and the second days [36,37]. There was a tendency for the total path length of the first trial of day 1 to be lower in KO mice (Fig 1E), suggestive of decreased exploratory activity, which is consistent with the previous report demonstrating that overexpression of NCS-1 promotes exploratory behavior in mice [17]. This may explain why the total path length on the first day was significantly lower in KO mice (Fig 1B). We also calculated the average speed for each training day, which was significantly reduced in KO mice, suggesting that motor activity is reduced in KO mice. However, data from the treadmill equipment suggested otherwise. We therefore concluded that the reduced average speed observed in KO mice may be an example of depressive-like behavior, which has also been described in previous reports [29], because the KO mice often floated with a stiff posture, which resulted in increased escape latency. Thus, our data demonstrate that KO mice exhibit cognitive defects, in accordance with the findings of previous studies that have used a variety of mouse models. These studies suggest that (1) NCS-1 overexpression in the dentate gyrus promotes synaptic transmission and acquisition of spatial memory [17]; and that (2) NCS-1 knock-down by siRNA or point mutation of NCS-1 harboring a destabilized NCS-1 protein results in deficiencies in exploration and learning function, as assessed using the object recognition test, hole-board test, and new frontier exploration test in mice [28]. Researchers have also reported that NCS-1 KO mice exhibit anxiety and depressive-like behavior, in addition to impaired non-aversive memory [29]. Taken together, these findings strongly reinforce the idea that NCS-1 is a critical regulator of spatial learning and memory function.

We also found significantly lower levels of BDNF in the cortex, hippocampus, basal ganglia (Fig 4A), and total forebrain (which includes all of these regions; Fig 4B) of KO mice. However, the difference was not statistically significant when comparing each specific region (p = 0.328, 0.132 and 0.078 for hippocampus, cortex and basal ganglia, respectively using Student’s *t*-test). Thus, NCS-1 appears to regulate BDNF levels in the mouse brain without regional specificity. Reduced BDNF levels in the KO mice may be an indirect consequence of generally lower brain activity, due to reduced exploration behavior [29]. If so, this may result in reduced BDNF production or accelerated degradation since neuronal activity is reported to regulate the transcription of BDNF gene [38]. Appropriate BDNF levels are essential for maintaining sufficient learning and memory function: Research has indicated that a genetic modification-induced increase in BDNF levels enhances learning and memory function [31], whereas down-regulation of BDNF impairs long-term potentiation [39]. In this way, BDNF may at least indirectly contribute to NCS-1-mediated spatial learning and memory functions (although a direct contribution may also exist via CaMKII, as described below). On the other hand, levels of another neurotrophic factor, GDNF, did not differ between the brains of WT and KO mice. We previously reported that exposure of neurons to GDNF in primary cultures increases levels of NCS-1 and improves neuronal survival under stressed conditions [19]. These results suggest that NCS-1 is a downstream target of GDNF, but not an upstream regulator of GDNF expression. These findings raise the interesting possibility that NCS-1 has a dual function as a positive regulator of memory/learning and neuronal survival under stress, as an upstream target of BDNF and a downstream target of GDNF, respectively.

There were no gross abnormalities in hippocampal structure, numbers of CA pyramidal or DG granule neurons, or increases in apoptosis in KO mice. These results suggest that the functional impairment observed in KO mice is not due to neuronal mal-development, atrophy, or neural cell loss. However, ultrastructural analysis using transmission electron microscopy revealed that the density of LDCVs decreased in the presynaptic terminals of CA1 hippocampal neurons, which may explain the decreases in levels of BDNF and dopamine observed in the brains of KO mice, as LDCVs are known to secrete both BDNF and dopamine. Although the intracellular mechanism of this phenomenon remains unclear at present, it is interesting to note the similarity between NCS-1 and calcium-dependent activator protein for secretion 2 (CAPS2), a dense-core vesicle-associated protein. CAPS2 promotes BDNF secretion [40], and its mutation is associated with autism and anxiety-like behavior [41]. NCS-1 deficiency also results in decreased levels of BDNF (present data) and causes anxiety and depressive-like behavior [29]. Therefore, it is possible that calcium-dependent signaling may be involved in regulating the effects of these two proteins via a common mechanism.

CaMKII is a multifunctional serine/threonine holoenzyme enriched in the hippocampus and is essential for memory formation and synaptic plasticity [42,43]. Indeed, long-term potentiation induction results in Ca^2+^ entry, which subsequently activates CaMKII. CaMKII then binds to NMDA-type glutamate receptors and potentiates AMPA-type glutamate receptors by phosphorylating their principal and auxiliary subunits, resulting in increased excitatory postsynaptic current [2]. In the present study, we observed that levels of phosphorylated CaMKII-α are significantly reduced in the KO brain under basal conditions. In addition, repeated application of HELP increased the levels of NCS-1 and phosphorylated CaMKII-α in the hippocampus with similar patterns. Furthermore, our preliminary experiments revealed that electrical tetanic stimulation (50 Hz, 2 s duration, at every 20 s for 5 min), which increases excitatory post synaptic current [44], resulted in increased cellular phosphorylated CaMKII-α levels concomitant with their translation to the nucleus (which is reported to occur by intracellular Ca^2+^ mobilization [13]) in cultured neurons of WT mice (S2 Fig). However, little translocation was observed in KO neurons, and overexpression of NCS-1 rescued the deficiency in KO neurons (S2 Fig). These results strongly suggest a close relationship between NCS-1 and CaMKII-α activation, thus implicating both in spatial learning and memory function. Regarding BDNF, we previously reported that HELP increased levels of BDNF in the hippocampus (and cortex) and significantly improved spatial learning and memory performance [33]. In the present study, we again observed that HELP significantly increases BDNF levels. Electric field stimulation increases BDNF levels via Ca^2+^-dependent signals, such as activation of voltage-gated Ca^2+^ channels [45], which also interact with NCS-1 to promote synaptic facilitation and neurotransmission [15,21,28]. Moreover, BDNF transcription is promoted by CaMKII-α activation [13]. Thus, we speculate that HELP may increase intracellular Ca^2+^ signals via an NCS-1-mediated pathway and consequently activate CaMKII-α, which in turn promotes BDNF transcription. This concept is supported by our previous report, which indicated that NCS-1 deficiency decreases Ca^2+^ signals and CaMKII activity in the heart [23]. However, since the pattern of BDNF increment is quite different from that of NCS-1 and phosphorylated CaMKII-α, other pathways independent of NCS-1 may exist to increase BDNF following HELP treatment.

NCS-1 interacts with dopamine D2 receptors in order to regulate dopamine signals [26]. The results of the present study indicate that levels of dopamine are decreased in the brains of KO mice, consistent with the findings of a recent study, which demonstrated that NCS-1 KO mice have significantly lower activity-dependent dopamine release in the core of the nucleus accumbens as well as reduced motivated behavior [30]. Dopaminergic signals and BDNF levels are largely intertwined. Stimulation of striatal neurons with dopamine results in enhanced BDNF production achieved through intracellular Ca^2+^ increases, which is followed by CaMKII-α activation [13]. These findings largely support the close relationship among NCS-1, dopamine, Ca^2+^ signaling, BDNF, and spatial learning and memory function. Abnormal activity of dopamine signaling has been implicated in several psychiatric and neurological disorders. Samples of the prefrontal cortex of patients with schizophrenia and bipolar disorder exhibit increased expression of NCS-1 [46]. These results provide additional support for the claim that NCS-1 activity may be associated with psychiatric symptoms. Moreover, NCS-1 is involved in cocaine addiction [47]. Clarification of NCS-1-related signaling pathways is critical for the development of pharmaceutical interventions aimed at treating psychological disorders, drug addiction, and presently irreversible disturbances of spatial learning and memory.

## Conclusions

Our data demonstrate that NCS-1-mediates spatial learning and memory function at least in part through activation of CaMKII-α signaling, which may directly or indirectly increase BDNF production.

## Supporting Information

S1 Fig Localization of neuronal calcium sensor-1 (NCS-1) in the mouse brain. High-magnification images of the cortex, midbrain, and cerebellum of 6-week-old wild-type (WT) and *Ncs1*^-/-^ (knock-out [KO]) mice subjected to immunohistochemistry for NCS-1 localization. The image enclosed in the square shows that NCS-1 is also expressed in axons. The nuclei are counterstained with hematoxylin and visualized in blue. Scale bars = 20 μm for cortex and midbrain, and 200 μm for cerebellum.
(PDF)

S2 Fig Expression levels and localization pattern of phosphorylated CaMKII-α with and without electrical stimulation in cultured neurons. Cerebral neurons obtained from WT and KO mice were cultured for 3 days, and then infected with adenovirus carrying NCS-1 or LacZ. For the next 24h, some neuron preparations were electrically stimulated (ES, 50 Hz, 2s duration, at every 20 s for 5 min). Neurons were fixed to examine the expression levels and localization pattern of phosphorylated CaMKII-α (P-CaMKII-α) with immunofluorescence confocal microscopy. (A and B): Representative images depicting that Ad-NCS-1 causes elevated levels and nuclear translocation of phosphorylated CaMKII induced by electrical stimulation in WT, but not KO neurons. Nuclei were visualized with DAPI. The scale bar is 10 μm. (C) Quantitative analysis of the average fluorescent intensity of data shown in panels A and B. Statistical testing with Two-way ANOVA shows significance when comparing the experimental conditions (DF = 1, F = 13.447, P = 0.0001), and when comparing groups (WT, KO and KO+NCS-1; DF = 2, F = 1.831, P = 0.175). (D) Quantitative analysis of the nuclear translocation of phosphorylated CaMKII. The fluorescence intensity of the nucleus was dividing by the fluorescent intensity in the rest of the cell (i.e. whole cells—nucleus) to obtain the relative fluorescent intensity (Two-way ANOVA, DF = 1, F = 5.061, P = 0.034 when comparing experimental conditions, and DF = 2, F = 5.772, P = 0.009 when comparing groups).
(PDF)
