# Supplementary figures and images for: Possible Signaling Pathways Mediating Neuronal Calcium Sensor-1-Dependent Spatial Learning and Memory in Mice
Source: PLoS One. 2017 Jan 25;12(1):e0170829. doi: 10.1371/journal.pone.0170829 (PMC5266288; doi:10.1371/journal.pone.0170829)

**S1 Fig**

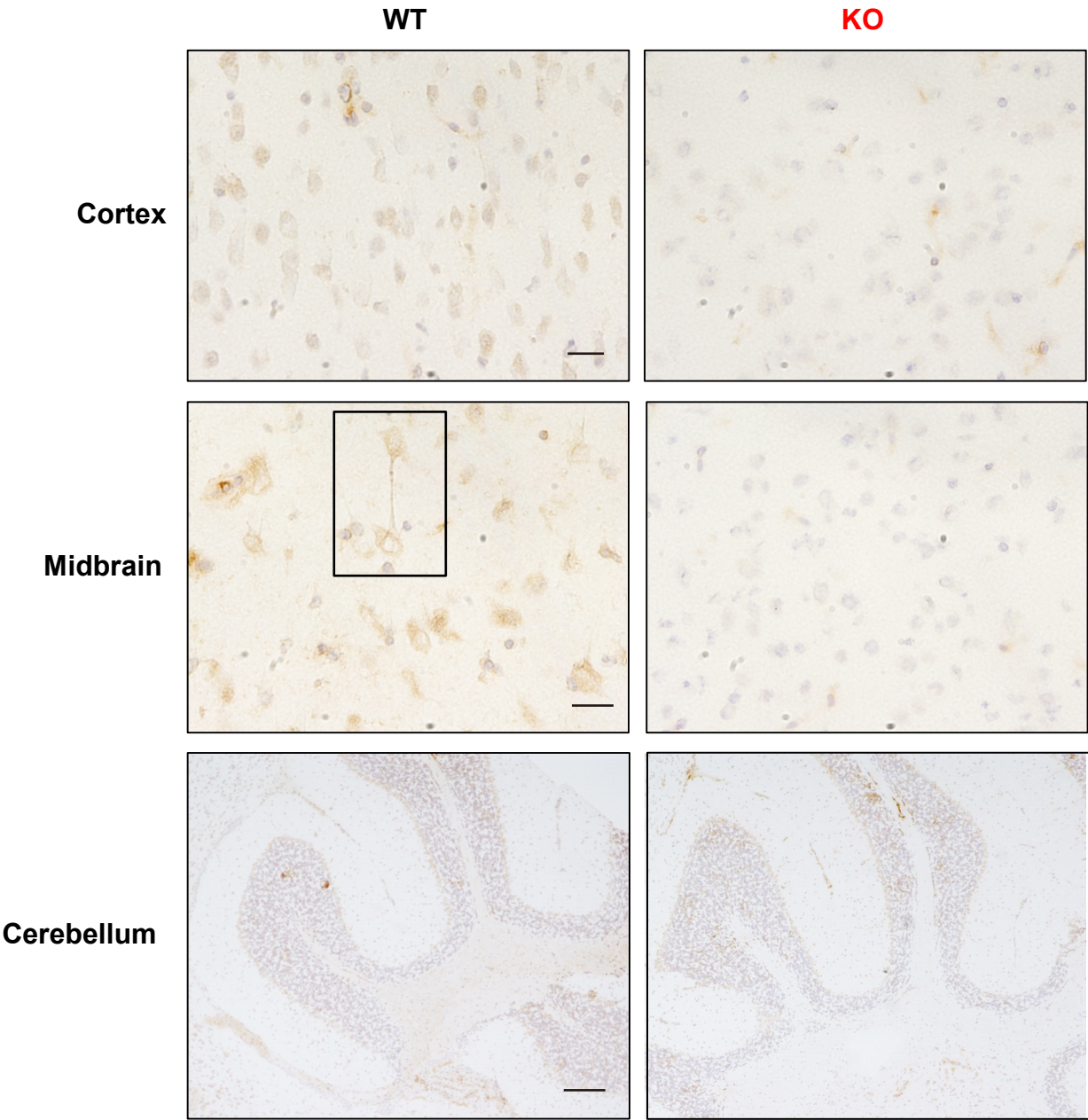

Supplement: S1 Fig — High-magnification images of the cortex, midbrain, and cerebellum of 6-week-old wild-type (WT) and Ncs1-/- (knock-out [KO]) mice subjected to immunohistochemistry for NCS-1 localization. The image enclosed in the square shows that NCS-1 is also expressed in axons. The nuclei are counterstained with hematoxylin and visualized in blue. Scale bars = 20 μm for cortex and midbrain, and 200 μm for cerebellum. (PDF) [file pone.0170829.s001.pdf]

# S2 Fig

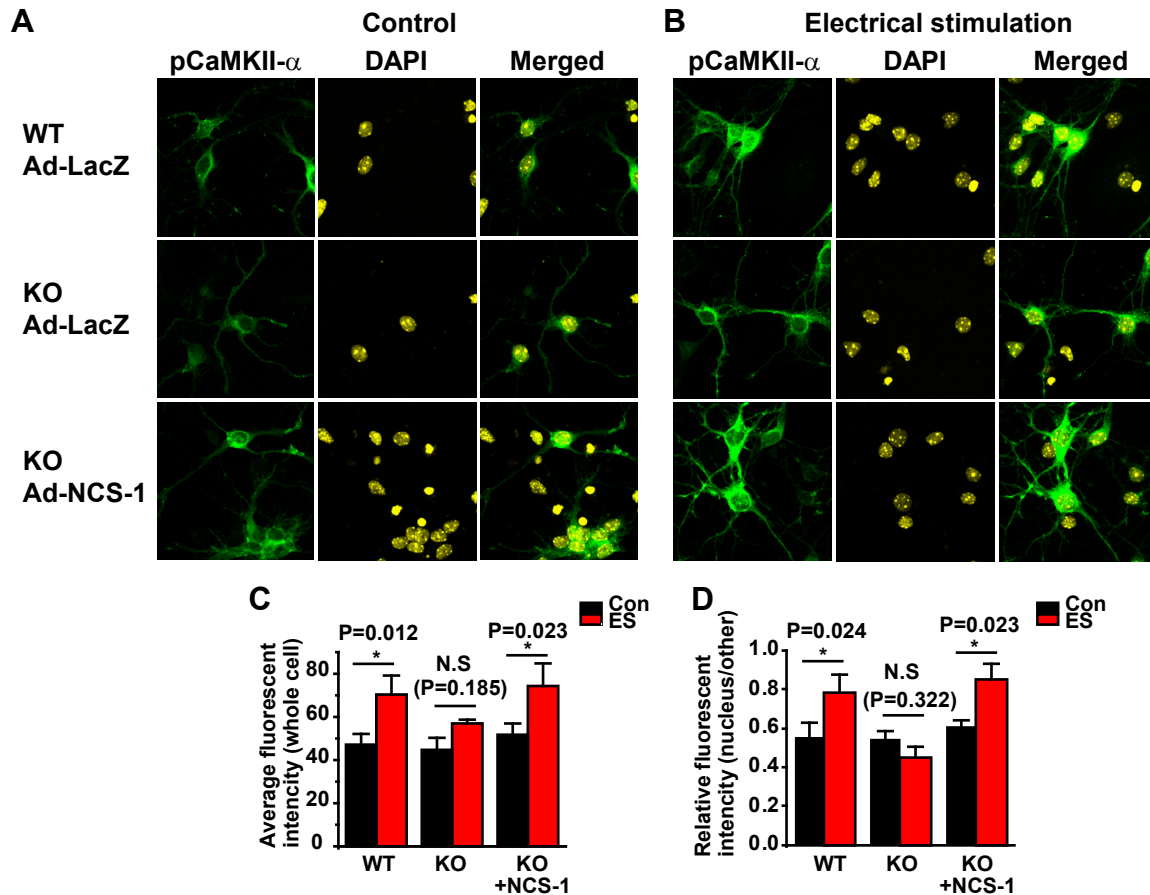

Supplement: S2 Fig — Cerebral neurons obtained from WT and KO mice were cultured for 3 days, and then infected with adenovirus carrying NCS-1 or LacZ. For the next 24h, some neuron preparations were electrically stimulated (ES, 50 Hz, 2s duration, at every 20 s for 5 min). Neurons were fixed to examine the expression levels and localization pattern of phosphorylated CaMKII-α (P-CaMKII-α) with immunofluorescence confocal microscopy. (A and B): Representative images depicting that Ad-NCS-1 causes elevated levels and nuclear translocation of phosphorylated CaMKII induced by electrical stimulation in WT, but not KO neurons. Nuclei were visualized with DAPI. The scale bar is 10 μm. (C) Quantitative analysis of the average fluorescent intensity of data shown in panels A and B. Statistical testing with Two-way ANOVA shows significance when comparing the experimental conditions (DF = 1, F = 13.447, P = 0.0001), and when comparing groups (WT, KO and KO+NCS-1; DF = 2, F = 1.831, P = 0.175). (D) Quantitative analysis of the nuclear translocation of phosphorylated CaMKII. The fluorescence intensity of the nucleus was dividing by the fluorescent intensity in the rest of the cell (i.e. whole cells—nucleus) to obtain the relative fluorescent intensity (Two-way ANOVA, DF = 1, F = 5.061, P = 0.034 when comparing experimental conditions, and DF = 2, F = 5.772, P = 0.009 when comparing groups). (PDF) [file pone.0170829.s002.pdf]
